# Laser-Supported Dual Energy X-Ray Absorptiometry (DXL) Compared to Conventional Absorptiometry (DXA) and to FRAX as Tools for Fracture Risk Assessments

**DOI:** 10.1371/journal.pone.0137535

**Published:** 2015-09-28

**Authors:** Hans Lundin, Faramarz Torabi, Maria Sääf, Lars-Erik Strender, Sven Nyren, Sven-Erik Johansson, Helena Salminen

**Affiliations:** 1 Department of Neurobiology, Care Sciences and Society, Karolinska Institutet, Stockholm, Sweden; 2 Department of Molecular Medicine and Surgery, Karolinska Institutet, Stockholm, Sweden; University of Hull, UNITED KINGDOM

## Abstract

Dual X-ray and Laser (DXL) adds a measure of the external thickness of the heel, measured by laser, to a conventional measurement of bone mineral density (BMD) of the calcaneus, using Dual energy X-ray Absorptiometry (DXA). The addition of heel thickness aims at a better separation of fatty tissue from bone than the standard method of DXA, which may mistake fatty tissue for bone and vice versa. The primary aim of this study was to evaluate whether DXL of the calcaneus can be used to assess the 10-year risk of fractures. Secondary aims were to compare the predictive ability of DXL with the two most established methods, Dual energy X-ray Absorptiometry (DXA) of the hip and spine and the WHO fracture risk assessment tool, FRAX. In 1999 a cohort of 388 elderly Swedish women (mean age 73.2 years) was examined with all three methods. Prospective fracture data was collected in 2010 from health care registers. One SD decrease in BMD of the heel resulted in an age-adjusted Hazard Ratio (HR) of 1.47 for a hip fracture (95% CI 1.09–1.98). Harrell’s C is the Cox regression counterpart of the Area Under Curve (AUC) of the Receiver Operating Characteristic (ROC) as a measure of predictive accuracy. Harrell’s C for BMD of the calcaneus was 0.65 for prediction of hip fractures. These results were not significantly different from those for BMD of the femoral neck or for FRAX. The HR for a hip fracture, for one SD decrease in BMD at the femoral neck, was 1.72 (95% CI 1.21–2.44. Harrell’s C was 0.67 for BMD at the femoral neck and 0.59 for FRAX. We conclude that DXL of the calcaneus could be a useful tool for fracture risk assessments.

## Introduction

Low-energy fractures lead to large costs for direct treatment of the fracture but often also to subsequent costs for help with activities of daily life, as the fractures often lead to functional impairment for the affected individuals. For example, only about one in three individuals reaches pre-fracture functional status after a hip fracture [1]. Effective preventive treatments are available. High-risk patients may benefit greatly from preventive interventions while low-risk patients may not. To decide which patients to offer fracture preventive treatments, it is therefore important to be able to estimate fracture risk accurately. Since low Bone Mineral Density (BMD) is a strong risk factor for fractures, a measurement of BMD is often included in a fracture risk assessment. The standard method for such a measurement is Dual energy X-ray Absorptiometry (DXA). The fracture risk increases approximately 1.5 to 2.6 fold for every decrease of one Standard Deviation (SD) in BMD from the age-standardized mean [2]. DXA is the recommended technique to establish the diagnosis of osteoporosis [3]. It is also the clinical standard to monitor effects of medical treatment aimed at fracture prevention, although changes in BMD have been shown to be unreliable in this respect [4,5], with the possible exception of treatment with Denosumab [6].

DXA of the hip and spine is included in most national guidelines as part of a fracture risk assessment [7–9].

There are, however, some disadvantages associated with the use of the conventional DXA device. It is costly, around $130,000, and because of its weight it is stationary and therefore needs to have a dedicated room. The device also needs to be operated by well-trained staff, e.g. to position the patient right on the table. Since DXA has been considered insufficient as a single predictor in a fracture risk assessment [10], several other methods for fracture prediction, alternative or supplemental to DXA, have been developed [11]. DXA of the femoral neck is the BMD measurement which is included in FRAX, the most widespread fracture prediction tool worldwide. The result of a FRAX calculation is the absolute ten-year fracture risk [12].

The ability of BMD at the calcaneus (heel bone) to predict fractures has been investigated with different techniques in previous studies:

The calcaneus was a better site for fracture prediction than the radius or lumbar spine in a study using Single X-ray Absorptiometry (SXA), [13]. Similar results have been shown when BMD of the calcaneus was measured with Single-Photon Absorptiometry (SPA) [14,15] or with DXA [16]. DXA may confuse bone with adipose tissue. The absorptive properties of adipose tissue differ and the amount of adipose tissue in the bone marrow varies with age. Therefore traditional DXA measurements of the hip and lumbar spine can have error margins as large as ±1 standard deviation in T-score (precision error plus accuracy error) [17].

### Studies on DXL

A cross-sectional comparison of DXL and DXA of the spine in our study population was published in 2005 [18]. The correlation between BMD of the calcaneus and the femoral neck was shown to be moderate, with a Pearson’s correlation coefficient of about 0.6. It was also concluded that it was risky to compare the DXL and DXA as measuring techniques based on T-scores since the T-scores were profoundly dependent on what reference population was used. DXA of the hip uses the NHANES III database [19] as reference whereas DXL has its own reference population [20]. BMD of the calcaneus measured with Dual X-ray and Laser (DXL) has shown a good predictive ability for hip fractures in another Swedish population of 4,398 women. There the Area Under Curve (AUC) for the Receiver Operating Characteristic (ROC) was 0.84 [21]. In a cross-sectional study on the discriminative ability for prevalent vertebral fractures of DXL and DXA, DXL was found to have a discriminative ability similar to that of DXA [22].

The primary aim of this study was to investigate whether BMD of the calcaneus, measured with DXL technology, could assess the ten-year risk of fractures. Secondary aims were to compare the predictive ability of DXL to the standard DXA of the hip and spine and to WHO’s fracture assessment tool FRAX. To our knowledge, this is the first study on ten-year fracture risk assessment with DXL.

## Material and Methods

### Population

**Fig 1:**
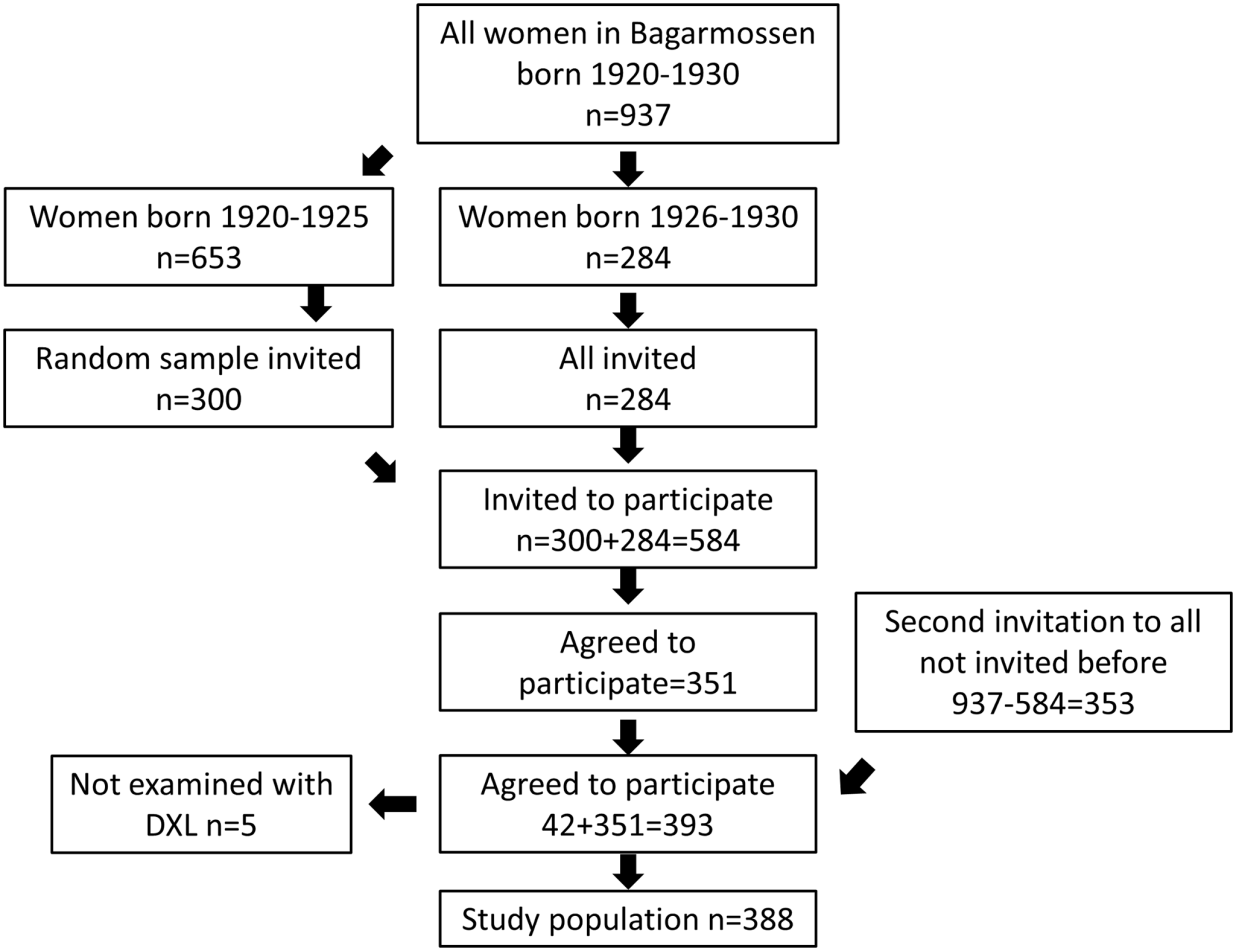
Flowchart of the participants in the study.

The population-based cohort study included 388 elderly women taking part in the PRIMOS (Primary Health Care and Osteoporosis) project. The inclusion criteria were: being a woman living in the area of Bagarmossen south of Stockholm, Sweden, and having been born between 1920 and 1930. The women had to be able to come to the primary health care center in Bagarmossen and to Karolinska University Hospital in Solna, 14 kilometers away from Bagarmossen. No other exclusion criteria were applied. The participants were sent written invitations followed up by a phone call from the person responsible for the study. Out of 937 eligible individuals, 388 were included (Fig 1).

The inclusion data was collected between March 1999 and February 2001. In 2010, fracture data was obtained from the Swedish National Board of Health and Welfare. Results of cross-sectional analyses of the BMD measurements within the PRIMOS population have been published in previous studies [18,23,24].

### DXA and DXL technologies

BMD was measured at the left calcaneus using a DEXA-T (Stille AB, Stockholm, Sweden) device. It uses fan beam configurated X-rays to measure BMD, and a laser beam to measure the distance between the skin surfaces of the opposing sides of the heel (i.e. the total thickness of the heel). This combined technique is called “Dual X-ray and Laser” (DXL). The updated version of the DEXA-T is the Calscan (Demetech, Stockholm, Sweden) which is the 4th generation of the DXL device. The longitudinal in-vivo precision (CV) of the Calscan has been estimated at 1.2% [25]. This precision is similar to that of conventional DXA machines measuring axial BMD at the femoral neck (CV 1.2%), and spine (CV 1.1%) [26].

The DXA measurements were conducted between March 1999 and February 2001 using Hologic QDR 4500 DXA equipment (Hologic, Waltham, Md., USA). Both hips were measured whenever possible but in the analyses in this study, only BMD of the left hip was used. Thus the BMD measurements of the hip and calcaneus came from the same leg, to eliminate the possible effect on BMD of differential loading on the left and right legs.

Kullenberg and colleagues have created a Swedish reference population for DXL consisting of 993 women between the ages of 15 and 85 [20]. That reference population was used to calculate the T-scores at the calcaneus in this study.

### Fracture outcomes

Follow-up data regarding fractures and mortality was obtained from the Swedish Board of Health and Welfare. The fractures were sorted into two groups:

1. “Major osteoporotic fractures” according to the definition in FRAX, i.e. fractures of the hip, vertebrae, humerus or distal radius caused by low energy trauma. This group of fractures includes the ICD-10 codes S72.x, S52.x, S32.x, S42.2, S42.3, S42.4, S22.0 and S22.1.
2. “Hip fractures” with the ICD-10 code S72.x

### Statistical analyses

Student’s t-test was used for comparison of means of normally distributed variables. For skewed distributions, the Mann-Whitney rank sum test was used. The two-tailed chi^2^ test was used for comparison of proportions except for when a group contained five or fewer observations, where Fisher’s exact test was used instead. In significance analysis of differences between the two lowest quartiles of BMD at the femoral neck and calcaneus, we analyzed the participants who belonged to only one of the two quartiles and not those included in both quartiles.

Comparisons regarding differences in time from inclusion to event were made using Cox regression. All Cox regression models were tested and accepted regarding Goodness of Fit and the Proportionality Assumption.

Concordance probability, Harrell’s C, was estimated from Martingale, Cox-Snell and deviance residuals.

Alpha was set at 0.05, and all analyses were performed with STATA 11.2 (StataCorp LP, Texas, USA).

### Ethical considerations

The study design and the procedure for obtaining informed consent was approved by the Regional Ethical Review Board at Karolinska Institutet, Stockholm, Sweden.

Since written informed consent was not required in the instructions from the Regional Ethical Review Board at the time for the vetting in 1999, verbal informed consent was obtained from all participants before inclusion. The fact that verbal and written information about the study had been given to the participant and that the participant had given her verbal consent to participate, was documented in her study protocol at the first visit.

## Results

In the study population of 388 women, 82 women (21%) had a major osteoporotic fracture during a mean follow-up time of 9.9 years. Of those 82 fractures 43 (53%) were hip fractures. This results in an incidence rate of 26 major osteoporotic fractures per 1000 person-time years at risk and 13 hip fractures per 1000 person-time years at risk.

Spearman’s correlation between BMD at the heel bone and at the femoral neck was 0.58 and between the corresponding T-scores 0.58 as well. Spearman’s correlation between femoral neck and lumbar spine BMD was 0.61.

**Fig 2:**
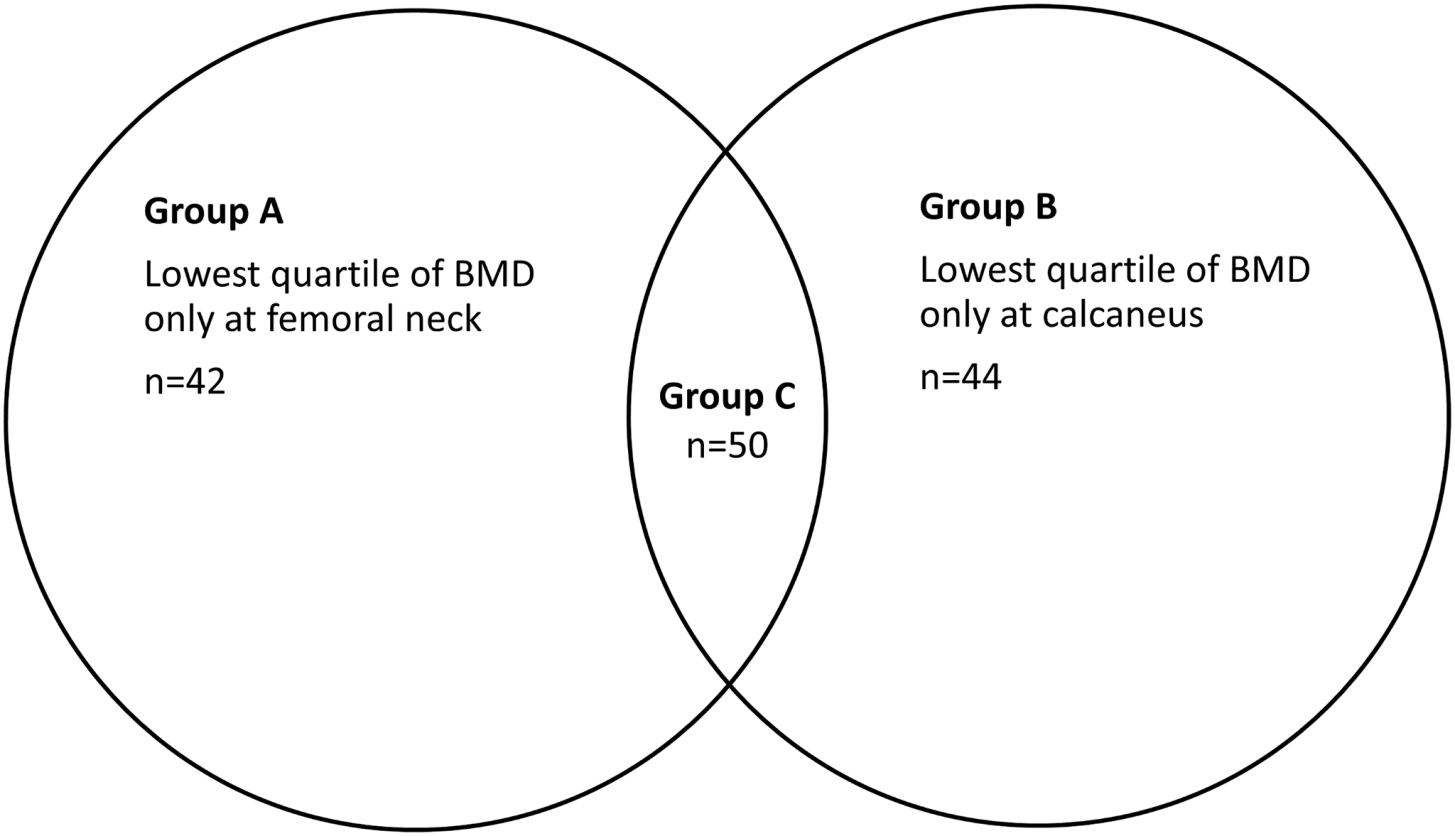
Venn diagram of the distribution of the participants between the lowest quartiles of BMD according to DXA of the femoral neck (n = 42+50 = 92) and DXL of the calcaneus (n = 44+50 = 94). Participants in group C are included in the lowest quartile at both sites.

Quartiles of BMD were created from the measurements at the calcaneus and from the femoral neck. About half of the lowest quartile of BMD at each site overlapped with the lowest quartile at the other site (Fig 2). Despite this lack of overlap in BMD, the baseline characteristics showed a similar fracture risk profile (Table 1). Group A, the participants which were in the lowest quartile of DXA-measured BMD but not in the lowest quartile of DXL-measured BMD were distributed like this: 62% were found in the second quartile of DXL-measured BMD, 33% in the third quartile and 5% in the quartile with the highest DXL-measured BMDs. Group B, the participants which were in the lowest quartile of DXL-measured BMD but not in the lowest quartile of DXA-measured BMD are distributed like this: 64% were found in the second quartile of DXA-measured BMD, 28% in the third quartile, and 8% in the quartile with the highest DXA-measured BMDs.

**Table 1:** Baseline characteristics with participants divided into groups according to quartiles of BMD at calcaneus and femoral neck. Groups A, B and C are the same as in Fig 2.

	Group A:The lowest quartile of BMD only at femoral neck	Group B:The lowest quartile of BMD only at calcaneus	Group C:The lowest quartile of BMD at both calcaneus and femoral neck	Group D:The highest three quartiles of BMD^a^ at calcaneus and/or femoral neck	p-value^a^ for Δ A-B = 0
**Number of participants**	42	44	50	246	n.a.
**Age**, mean (SD)	74.1 (3.25)	73.7 (3.13)	74.3 (2.94)	73.2 (2.61)	0.58
**BMI**, mean (SD)	25.1 (4.88)	25.0 (3.82)	23.6 (3.15)	27.7 (4.24)	0.86
**FRAX hip** ^b^, % (SD)	14.9 (10.0)	13.8 (10.9)	18.4 (11.0)	9.3 (6.05)	0.63
**FRAX major osteoporotic** ^c^, % (SD)	28.0 (11.6)	26.8 (11.3)	32.6 (11.6)	21.5 (8.58)	0.64
**Previous fracture after age 50** (%)	13 (31.0%)	20 (45.4%)	25 (50%)	60 (24.4%)	0.167
**Mother fractured hip** (%)	6 (14.3%)	5 (11.4%)	7 (14.0%)	25 (10.1%)	0.76
**Current smoking** (%)	10 (23.9%)	10 (22.7%)	10 (20.0%)	24 (9.8%)	1.00
**Diabetes** (%)	3 (7.1%)	1 (2.27%)	1 (2.0%)	24 (9.8%)	0.355

^a^. p-value for no difference between group A and group B

^b^. 10-year absolute risk of a hip fracture according to FRAX including BMD of the femoral neck

^c^. 10-year absolute risk of a major osteoporotic fracture (hip, vertebral, humerus or radius) according to FRAX including BMD of the femoral neck

There was a trend toward a higher prevalence of previous fractures after age 50 in the lowest quartile of BMD at the calcaneal (group A) compared to the lowest quartile at the femoral neck (group B), p = 0.167. The prevalence of diabetes mellitus type 1 or 2 was five times higher (p = 0.093) in the group with BMD in the highest three quartiles at the calcaneus and/or at the femoral neck (group D) than in the group with BMD in the lowest quartile at both the calcaneus and the femoral neck (group C).

The group with BMD in the lowest quartile at both the calcaneus and the femoral neck (group C) had twice as high prevalence of both smoking (p = 0.038) and a previous fracture (p<0.001) after age 50, as the group with BMD in the highest three quartiles at the calcaneus and/or at the femoral neck (group D). However, adjustment for presence of diabetes or smoking and previous fracture in the Cox regression models presented in tables 2 and 3, did not alter any of the results significantly.

**Table 2:** Age-adjusted Hazard Ratios (HR) for fractures for one Standard Deviation (SD) decrease in BMD at calcaneus and femoral neck.

	Hip fracture	Major osteoporotic fracture
**–1 SD in BMD left calcaneus, HR (95% CI)**	1.47 (1.09–1.98) p = 0.011	1.37 (1.11–1.70) p = 0.004
**–1 SD in BMD femoral neck, HR (95% CI)**	1.72 (1.21–2.44) p = 0.002	1.30 (1.02–1.64) p = 0.033
**–1 SD in BMD total hip, HR (95% CI)**	1.58 (1.16–2.16) p = 0.004	1.32 (1.06–1.65) p = 0.015
**–1 SD in BMD lumbar spine, HR (95% CI)**	1.00 (0.75–1.35) p = 0.976	1.14 (0.91–1.42) p = 0.246

**Table 3:** Age-adjusted Hazard Ratios (HR) for a hip fracture, lowest quartile of BMD compared to the higher three quartiles of BMD, measured with DXL of the calcaneus or DXA of the femoral neck.

	Highest three quartiles of BMD	Lowest quartile of BMD
**DXL calcaneus, HR (95% CI)**	1.0 (reference group)	2.39 (1.30–4.39) p = 0.005
**DXA femoral neck, HR (95% CI)**	1.0 (reference group)	2.35 (1.26–4.36) p = 0.007
**DXA total hip, HR (95% CI)**	1.0 (reference group)	2.41 (1.30–4.45) p = 0.005
**DXA lumbar spine, HR (95% CI)**	1.0 (reference group)	0.76 (0.36–1.58) p = 0.456

The age-adjusted HR for a hip fracture was 1.47 (95% CI 1.09–1.98) for 1 SD decrease in BMD at the calcaneus. For comparison, 1 SD decrease in BMD at the femoral neck resulted in an age-adjusted HR for a hip fracture of 1.72 (95% CI 1.21–2.44) (Table 2).

The HRs for 1 SD decrease in BMD were statistically significant and had overlapping confidence intervals at all sites for both hip fractures and major osteoporotic fractures. The exception was the lumbar spine where the HR was non-significant.

The HRs for a hip fracture were similar and statistically significant at all sites except for the lumbar spine, when those included in the lowest quartile of BMD were compared to the three higher quartiles of BMD (table 3). T-scores were not used for comparison of DXL and DXA since T-scores are not only dependent on the measuring technique, but also on the reference population.

Harrell’s C is the equivalent to an Area Under Curve (AUC) value for a Receiver Operating Characteristic (ROC) used with logistic regression models but it is designed for Cox regression models and thus takes time-to-event into account.

Harrell’s C for a hip fracture was 0.61 for BMD of the calcaneus, 0.66 for BMD of the femoral neck and 0.59 for FRAX including BMD of the femoral neck.

When logistic regression was used instead of Cox regression, the AUC was 0.61 for BMD of the heel bone, 0.65 for BMD of the femoral neck and for FRAX including BMD of the femoral neck.

## Discussion

In our population of elderly women, DXL of the calcaneus selected a high-risk quartile with very similar characteristics to that selected by DXA of the femoral neck. The two techniques also seemed to be equally strong as fracture predictors since they had overlapping confidence intervals for the HR for fractures for 1 SD decrease in BMD. There was likewise no difference in predictive accuracy between the two methods as determined by Harrell’s C or AUC. The AUC for BMD at the calcaneus was 0.7 for a hip fracture in a study by Cummings and colleagues [16] which is similar to the AUC of 0.61 found in our study. A much higher age-adjusted AUC for hip fractures of 0.84 was found in a study by Brismar and colleagues [21]. Perhaps the explanation for this could be that the participants in our study were all elderly and that we probably had different inclusion criteria from those of Brismar and colleagues. In a study by Muschitz and colleagues the AUC for prevalent vertebral fractures was 0.665 was similar to the AUC for hip fractures found in our study.

The HR for a hip fracture for 1 SD decrease in BMD at the calcaneus found in our study, 1.47 (1.09–1.98), is not significantly different from the results in a study from Cummings and colleagues [14] where the corresponding age-adjusted RR was 1.66 (95% CI 1.22–2.26), since the confidence intervals overlap. In a meta-analysis published in 1996 by Marshall and colleagues, all prospective cohort studies published between 1985 and 1994 were considered [2]. It was concluded that all sites of measurement of BMD (hip, lumbar spine, distal and proximal radius and calcaneus) had similar predictive abilities for future fractures.

In our study, it is impossible to discriminate between “true” differences in BMD between skeletal sites and differences in the BMD caused by differences between the DXA and DXL techniques. To make such a distinction, one would have to measure BMD with both techniques in the same individuals at the same skeletal site. The modest correlation between BMD at the calcaneus and the femoral neck in our study is in agreement with findings in previous studies and it is not different from the degree of correlation found between hip and spine BMD when both are measured with DXA. The differences in BMD between DXA of the hip and DXL of the calcaneus could be true but still not have such an important clinical implication. Both DXA and DXL techniques are intended to be tools for risk stratification and as such they could be interchangeable, according to our study. The reason that DXA and DXL can have similar predictive abilities although there identify different high risk individuals (Fig 2) is probably that they both have only moderate sensitivity. This explanation can be supported by the AUCs which are moderately high. As an example, if two different binary tests (results could only be positive or negative) each had a sensitivity of 60% (true positives) for hip fractures, there could be as little as 50% of the positives in each group that were positive in the other test as well. Still, there would then be 5% of those who would have a hip fracture later that were not identified as positive by either of the tests.

Strengths in our study are that it is population-based, that no one was lost to follow-up and that it is conducted within the largest high-risk group in society, that is, elderly women. A limitation of our study is the inclusion criteria of being mobile, since this probably selects the healthiest individuals from the population as participants.

In conclusion, in our population of elderly women, BMD of the calcaneus measured with DXL had a fracture-predictive ability similar to BMD of the femoral neck measured with DXA, and to FRAX. Since the DXL equipment is also less costly, easier to operate and mobile, DXL of the calcaneus could be a useful tool as part of a clinical fracture risk assessment if DXA equipment is not readily available to the patient.
